# Assessment of Various Factors Contributing to Unfavourable Oral Health Care Seeking Behaviour: A Cross-Sectional Study

**DOI:** 10.7759/cureus.53037

**Published:** 2024-01-27

**Authors:** Priya Tharshini, Leena Selvamary, Aswath Narayanan, Ramesh Kumar S.G, Sujatha A

**Affiliations:** 1 Department of Public Health Dentistry, MP Smile Care, Coimbatore, IND; 2 Department of Public Health dentistry, Tamil Nadu Government Dental College and Hospital, Chennai, IND; 3 Department of Public Health Dentistry, The Tamil Nadu Dr.M.G.R. Medical University, Chennai, IND; 4 Department of Public Health Dentistry, Tamil Nadu Government Dental College and Hospital, Chennai, IND

**Keywords:** health care access, oral health care, dental health services, communication barriers, utilization

## Abstract

Background: Access to effective oral health care is crucial for a good quality of life. Unfortunately, many individuals face barriers in accessing the necessary oral health care services. By examining various factors, we can gain a better understanding of the challenges faced by the population and work towards improving oral health care outcomes. This study aims to assess the various factors contributing to unfavorable oral healthcare-seeking behavior in Chennai city.

Materials and methods: A cross-sectional study was conducted in Chennai city involving six hundred and twenty-four individuals from the general population from 12 wards by lottery method. The data collection process involved interviews using a pre-designed questionnaire, through which demographic information was gathered. The Penchansky and Thomas model was employed to assess barriers in service utilization. All completed questionnaires were included in the data analysis, which was performed using SPSS version 20.

Results: In the present study, among the various dimensions of access suggested by Penchansky and Thomas, the major reason for unfavorable oral healthcare-seeking behavior was accommodation (54.8%), followed by affordability (20.2%), accessibility (5.6%), acceptability (4.4%), and availability (1.1%). The other reasons that contributed were sociocultural factors (26.4%), lack of awareness (20.8%), and psychosocial factors (11.8%).

Conclusion: The present study highlights lack of time as the major factor contributing to unfavourable oral healthcare-seeking behaviour in an urban population. This finding contrasts with previous studies that have focussed on the lack of awareness about dental diseases and the high cost of dental treatment in rural areas.

## Introduction

India, being a developing country, is facing numerous challenges in rendering oral health care to its population [1]. Recent statistics reveal that there are 329 dental colleges in the country resulting in a dentist-to-population ratio of 1:9992 [2]. Despite this significant increase in dental colleges and a reduction in the dentist population ratio, the main challenge lies in the unequal distribution of oral health care services. Approximately 80% of the population resides in rural areas, where there is a severe shortage of qualified dental surgeons. Unfortunately, most young graduates prefer to establish their clinics in larger towns or metropolitan cities due to the attractive infrastructure and amenities available. Consequently, this leads to a concentration of dental surgeons in urban areas, where only 20% of the population resides, resulting in social injustice as defined by the inverse care law [3].

Tamil Nadu, the recipient of the best administrative award from the Government of India in 2019, has a health care system that mirrors the national trends. The growth of dental colleges in the state has been slow since 1980, but there was a notable increase with 14 new dental colleges between 2001 and 2010. Currently, there are 32 dental colleges in Tamil Nadu, resulting in a dentist population ratio of one dentist for every 3666 people [4]. However, there is a significant geographic imbalance in the distribution of dental colleges, with a focus on urban areas. Chennai, known as India's health capital, is home to 18 dental colleges, including one government dental college and 17 private institutions.

In this era, significant advancements in technology have greatly improved access to oral health care. Additionally, there has been an increased awareness of oral health, leading to better oral health-seeking behavior. These improvements are more prevalent in urban areas compared to rural areas. The availability of a larger workforce, due to the increase in dental colleges and a decrease in the dentist population ratio, has contributed to the availability of oral health care services. Furthermore, administratively sound policies have been implemented to break down barriers to access, such as affordability and transportation. Free and subsidized oral health care services are provided to address the issue of affordability, while public transport systems ensure greater accessibility.

Despite efforts to address barriers in urban areas, a significant number of people still do not seek basic oral health care or attend dental appointments regularly. According to Bommireddy et al., utilization is not just the willingness of people to seek care, but the actual attendance at the site of delivery of healthcare services to receive care [5]. This lack of utilization contributes to the persistence of oral diseases in these areas.

Previous scientific studies have focused on assessing barriers to oral health care utilization in primary health centres [6], community health centres [7], and rural areas [8], where access to dental care is limited. These studies have identified common barriers such as fear of dental treatment, lack of awareness about dental diseases, high treatment costs, and long distances to dental care providers. However, there is limited research on the utilisation of oral health care services in metropolitan cities where access to dental care is considered adequate.

To address this gap, the present study aims to determine the various factors contributing to an individual not seeking oral health care in an urban area through a door-to-door survey method in Chennai city. This research will provide valuable insights into the barriers faced by urban communities in accessing dental care and help inform strategies to improve oral health care utilisation in metropolitan areas.

The objectives of the study were to prepare an instrument (questionnaire) to assess factors contributing to unfavourable oral healthcare-seeking behaviour, to get an insight into the knowledge and attitude of their dental health problem using a questionnaire, and to identify the various factors like demographic, behavioural, socio-economic, cultural and epidemiological factors which hinder an individual from availing oral health care using a questionnaire.

## Materials and methods

Study design and setting

A cross-sectional study was designed to assess the various factors contributing to unfavourable oral health care-seeking behaviour among the General Population residing in Chennai City. The duration of the study was 6 months. The study was presented using STROBE guidelines.

Study Participants

Inclusion Criteria

The general population residing in Chennai City aged above 18 years

Exclusion Criteria

The exclusion criteria included subjects with mental disorders affecting communication and memory function, subjects with hearing and speech disability, and subjects who were severely debilitated at the time of assessment.

Sampling Procedure

Participants satisfying the predesigned selection criteria were selected by multistage random sampling. The Greater Corporation of Chennai is divided into three regions (North Chennai, Central Chennai, and South Chennai). From each region, two zones were selected (Total= six zones). Then, from each of the selected zones, two wards were selected (Total= 12 wards) by using the lottery method at each stage. Finally, from each selected ward, 52 study participants were recruited (Total= 624), by the KISH grid [9].

Sample Size

Sample size was estimated by OpenEpi, Version 3 open source calculator software with the formula n = [DEFF*Np(1-p)]/ [(d2/Z21-α/2*(N-1)+p*(1-p)]. The optimum sample size for the present study for a 95% confidence level was estimated to be 614. With the small adjustments for the non-response rate, 624 participants were recruited.

Study variables

The various variables that were used to assess the barriers to the utilization of oral health care services were the determinants of access such as availability, accessibility, affordability, acceptability and accommodation, awareness, psychosocial factors, and sociocultural factors among the general public residing in Chennai city.

Data sources/measurement

An ethical clearance certificate was obtained from the Institutional Review Board. The selected participants were clearly explained about the purpose of the study and assured about the confidentiality of their identity. Written informed consent was obtained from each participant prior to data collection. Data collection was done by interview method using a predesigned and pre-tested structured questionnaire.

The bilingually designed questionnaire (English and Tamil) consisted of 26 questions, which assessed the knowledge and attitude of their Dental problem, utilization, and barriers for seeking dental care and symptoms relating to dental diseases. To ensure the validity of the questionnaire, a panel of experts tested its face validity and content validity, with a content validity index (S-CVI) estimated at 0.95. The reliability of the questionnaire was tested with a test-pre-test method with a sample of 30 and was estimated as 0.92 (Cronbach’s alpha). The questionnaire were marked clearly through the interview method and coded and tabulated in Microsoft Excel 2010.

The data were analyzed using SPSS software (Version 22.0, 2013). Descriptive statistics were performed to characterize the sample and demonstrate the various factors contributing to unfavourable oral healthcare-seeking behavior. A model was created based on Thorpe et al. [10] to identify the various patterns of barriers in seeking medical care, and accordingly, the class I group was availability/accessibility/acceptability accounted for a minimal percentage. The Class II group constituted affordability, the Class III group with accommodation, and the Class IV group as combined or severe barriers. Multinomial logistic regression was used to calculate the relative risk ratios and adjusted relative risk ratios between the variables

## Results

The sample population consisted of 624 individuals, with an equal distribution of males and females. The largest group of respondents fell within the age range of 30 to 55 years, accounting for 54.4% of the population. The religious predominance composition of the study aligned with the data obtained from the 2011 census.

A majority of the participants (62.3%) were aware of the impact of general health on oral health. Table 1 displays the awareness of various oral diseases observed in the study population.

**Table 1:** Frequency distribution of awareness of various oral diseases

S.No.	Variables	N (%)
1.	General health affects oral health	389 (62.3)
	General health does not affect oral health	116 (18.5)
	General health may affect oral health	7 (1.1)
	Don’t know	112 (17.9)
2.	Decayed teeth	604 (96.7)
	Gum diseases	459 (73.5)
	Crooked teeth	41 (6.5)
	Discoloured teeth	56 (8.9)
	Mottled enamel	14 (2.2)
	Broken teeth	82 (13.1)
	Broken jaws	13 (2.0)
	Ulcers in the mouth	125 (20.0)
	Oral tumours	298 (47.7)
	Jaw joint problems	14 (2.2)
	I do not know	20 (3.2)
Dental Caries		
3.	White chalky spots	27 (4.3)
	Blackening on teeth	111 (17.7)
	Visible holes or pits	21 (3.3)
	Food getting stuck in between teeth	20 (3.2)
	Bad breath	34 (5.4)
	Teeth sensitive	104 (16.6)
	Toothache	35 (5.6)
	Pain while chewing	7 (1.1)
Periodontitis		
4.	Bad breath	56 (8.9)
	Swollen bright red gums	56 (8.9)
	Bleeding gums	12 (1.9)
	Painful gums	20 (3.2)
	Shaking teeth	42 (6.7)
	Loose teeth don’t fit on biting	20 (3.2)
	Tooth loss	105 (16.8)
Oral Cancer		
5.	White patches in the mouth	7 (1.1)
	Burning sensation in the mouth	7 (1.1)
Malocclusion		
6.	Habit of placing tongue in between teeth	7 (1.1)
	Biting nails	7 (1.1)
	Improper alignment of teeth	20 (3.2)
Fracture of teeth/jaws		
7.	Broken tooth due to injury	7 (1.1)
	Shaking of tooth	7 (1.1)
	Discolouration of tooth	14 (2.2)

Despite three-quarters (76.7%) of the participants recognising the perceived need for oral care, 10% admitted to never visiting a dentist, regardless of symptoms (Table 2).

**Table 2:** Visit to dental surgeon after the appearance of symptoms

Dental visit	N (%)
Immediately	358 (57.3)
I fix particular time for the symptoms to subside by itself	161 (25.8)
Do self-care home remedies as suggested by others or by the knowledge acquired by Internet	35 (5.6)
Until my Friends/Relatives force me	7 (1.1)
I will never visit a dentist because I can tolerate whatever symptom it is	63 (10.0)

**Figure 1:**
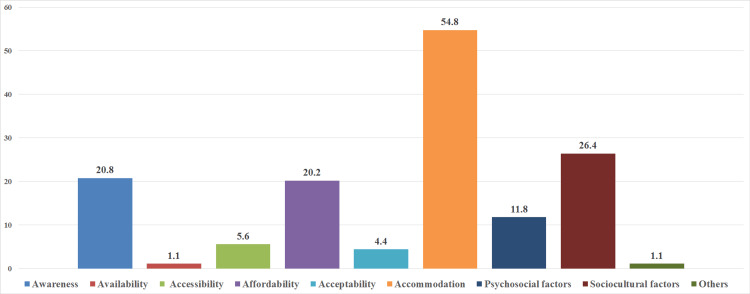
Various factors contributing to unfavourable oral healthcare seeking behaviour

The factors contributing to this unfavorable behavior varied, with a majority of respondents (51.2%) citing lack of time as the primary barrier to utilizing oral health services (Table 3, Figure 1).

**Table 3:** Frequency distribution of various factors contributing to unfavourable oral health care seeking behaviour

Variables	Reasons	N (%)
Awareness	I do not know that I have a dental problem	96 (15.3)
	Teeth are not at the top of the priority list	34 (5.4)
Availability	Dental service providers are not available nearby	7 (1.1)
Accessibility	Lack of proper transport	11 (1.7)
	Require assistance for transporting	8 (1.2)
	I do not have anybody to accompany me to the Dentist	16 (2.5)
Affordability	Financial problems (high cost of treatment)	34 (5.4)
	I do not have medical/ dental insurance	21 (3.3)
	Loss of one day's wages	71 (11.3)
Acceptability	I was not satisfied with the Dentist’s treatment	9 (1.4)
	Self-medication by over-the-counter drugs/ home remedies	19 (3.04)
Accommodation	Lack of time/ Leave	320(51.2)
	Dentists are busy and require long waiting hours	21(3.3)
	Requirement of multiple appointments for the treatment	2 (0.32)
Psychosocial factors	Fear for dentist	7 (1.1)
	Fear of dental treatment (pain of injection, sound of the drill, sitting in dental chair, dental environment, fear of white coat)	49 (7.8)
	Fear of quality of dental treatment	8 (1.2)
	Fear that treatment might lead to some other problems	10 (1.6)
Sociocultural factors	The dental problem does not interfere with my day-to-day activities	103(16.5)
	Sometimes other problems become overwhelming and self care unimportant	14 (2.2)
	I am not bothered because my teeth will be lost anyway	48 (7.6)
Others	No pain	7 (1.1)

The study population was divided into four classes based on the previous perceived barrier study done by Thorpe et al., and multinominal logistic regression between the variables was carried out [11]. Table 4 depicts that for every rise in the level of socioeconomic class, there was a two-fold increase in the accommodation barrier (2.211).

**Table 4:** Multinomial logistic regression predicting barrier class membership for Class II. NS- Non – Significant; Class I- Minimal barrier (Availability, Accessibility, Acceptability); Class II- Affordability; Class III – Accommodation; Class IV- Combined

Predisposing, Enabling barriers	Adjusted Relative risk ratio		
	Class II vs Class I	Class III vs Class I	Class IV vs Class I
Age	1.045 (0.995-1.097)	NS	2.411 (1.82-3.001)
Sex	NS	NS	NS
SES	NS	2.211 (1.78-3.86)	1.806 (1.061-3.073)
Type of Housing	1.99 (0.320-1.037)	NS	2.84 (1.88-4.82)
Religion	NS	NS	NS

## Discussion

Oral health is a key indicator of overall health, well-being, and quality of life. WHO defines oral health as “a state of being free from chronic mouth and facial pain, oral and throat cancer, oral infection and sores, periodontal disease, tooth decay, tooth loss, and other diseases and disorders that limit an individual’s capacity in biting, chewing, smiling, speaking and psychosocial wellbeing [11]. Oral health is important in the economic and social development of the country. Despite adequate development and urbanisation, oral health problems are still existing as a burden in urban cities. Therefore, this present study aimed to assess the various factors contributing to an individual not seeking oral health care among the residents of metropolitan cities like Chennai.

Access to healthcare is a critical factor in ensuring that patients receive appropriate care. Penchansky and Thomas emphasise that access enables patients to receive the right care from the right provider at the right time and in the right place [12]. Various dimensions influence access to healthcare services, including accessibility, availability, acceptability, affordability, and adequacy (accommodation) in service design, implementation, and evaluation [13]. These dimensions play a vital role in determining the extent to which individuals can access the care they need.

In the present study, the preeminent factor contributing to the unfavourable oral healthcare-seeking behaviour was Penchansky and Thomas's accommodation dimension, accounting for 54.8%. McKinslay's socio-cultural factors followed closely behind, accounting for 26.4%. Emily Saruman's awareness dimension was also found to be a contributing factor, representing 20.8% [13]. Additionally, affordability was identified as a predisposing factor in 20.2%, while psychosocial factors played a role in 11.8%.

Accommodation emerged as the primary reason for not seeking dental care in this study, with "lack of time/lack of leave" being reported by 51.2% of participants. This finding is consistent with previous studies conducted by Al Shammeri et al. [14], Poudyal et al. [15], and Fotedar et al. [16]. It is worth noting that 94.5% of participants were employed, due to which they refrained from seeking oral health care.

Another significant factor influencing access to dental care was the socio-cultural aspect. A notable rationale mentioned by participants was that "the dental problem does not interfere with my day-to-day activities," accounting for 16.5% of responses. This suggests that individuals only consider visiting a dental surgeon when they are symptomatic, compromising their overall quality of life, despite being aware of the importance of regular dental check-ups.

Lack of awareness also played a role, with 15.3% of participants stating that “I do not know that I have a dental problem”. Additionally, a proportion of the population expressed "I don't give priority to the teeth as the teeth are lost anyway." These findings indicate a minimal concern for oral health among this proportion of the population.

In accordance with previous studies by Nagarjuna P et al. [7] and Devaraj C et al. [17], the loss of one day's wages (11.3%) and high cost of dental treatment (5.4%) were reported as factors contributing to limited access to oral health care. Additionally, Fotedar S et al. [16] found that seeking oral health care only when experiencing pain was prevalent in our study as well. The majority of participants in our study belonged to the upper-lower class (57%), suggesting that affordability barriers may hinder immediate access to oral health care.

Following the affordability barrier, the other reason reported was “fear of dental treatment”, i.e. due to the pain of injection, the sound of the drill, sitting in a dental chair, the dental environment, and fear of white coat (8.9%). Moreover, the majority of the respondents felt that “teeth were not at the top of my priority list”, which demonstrates the underutilization of services. “Lack of dental insurance” also hindered individuals from obtaining dental care. Since the majority of health insurance covers only general health and not dental health, lack of dental insurance prevented them from seeking dental care.

The present study results suggest that creating more awareness regarding the utilization of oral health services is crucial. To address and minimize health care barriers, it is important to maintain proper appointment schedules, reduce waiting hours, minimize multiple appointments, and ensure promptness of health care providers. Fear-related barriers can be minimized through desensitization i.e. starting with less painful procedures and gradually progressing to more painful ones. Additionally, psychosocial and sociocultural factors are intertwined, and deep-rooted cultural factors should be thoroughly analysed within the community to eliminate unfavourable cultural factors. Educating the community about good oral health and motivating them to utilise the services can lead to an overall healthy, socially, and economically productive life.

The study's major strength lies in its larger sample size, allowing for more robust and reliable findings. Additionally, unlike the majority of studies that were hospital-based, this study was conducted at the community level through door-to-door interviews, enabling the results to be generalised to the entire population. Furthermore, the use of the KISH grid for participant recruitment minimised the risk of selection bias, while the interview method employed reduced non-response bias compared to self-administered questionnaires.

However, it is important to acknowledge the limitations of the study. The data collected was subjective, relying on an individual's perceptions of dental health and illness. This introduces the possibility of bias influenced by factors such as knowledge, attitudes, and cultural beliefs surrounding dental health. Additionally, participants may have answered questions in a manner that they perceived as socially desirable, potentially leading to social desirability bias.

## Conclusions

In the present study, the major factors contributing to unfavourable oral health care-seeking behaviour in the urban population are lack of time, dental problems not interfering with daily activities, lack of awareness about their own dental problems, loss of wages, and fear of dental treatment. However, professionals and the government can take necessary steps to improve oral health care by reinforcing existing education, creating more awareness, and modifying oral health programs and policies. Oral health should be incorporated into all health programs, especially general health programs.
